# The Association Between Pre-morbid Frailty and Discontinuation of Treatment Within 24 Hours in Critically Ill Older Adults Triaged to a Tertiary Care Emergency Department: A Retrospective Cohort Study

**DOI:** 10.7759/cureus.77222

**Published:** 2025-01-10

**Authors:** Atsuhito Tanaka, Ji Young Huh, Yoshinori Matsuoka, Koichi Ariyoshi

**Affiliations:** 1 Department of Emergency and Critical Care Medicine, Tokyo Bay Urayasu Ichikawa Medical Center, Chiba, JPN; 2 Department of Emergency Medicine, Kobe City Medical Center General Hospital, Kobe, JPN

**Keywords:** advanced care planning, clinical frailty scale, goal of care, pre-hospital intervention, withdrawal of care

## Abstract

Background

Some critically ill older adults would rather receive palliative than intensive care if their functional capacity and quality of life are compromised. Therefore, careful goals-of-care conversation is vital in frail older adults who are critically ill if there is no advance care planning before arrival at the emergency department. We considered that pre-morbid Clinical Frailty Scale (CFS) scores could be a potent factor in prioritizing such conversations if higher numbers were associated with increased withdrawal of treatment in these patients. This study aimed to investigate the association between pre-morbid frailty and discontinuation of treatment within 24 hours in older adults in Kobe, Japan.

Methodology

This retrospective, observational study was conducted among patients aged 65 and over who were triaged as needing tertiary care to our emergency department with subsequent admission for further treatment. The primary outcome was withdrawal of treatment within 24 hours. We examined the association between CFS and withdrawal of treatment using a multivariable logistic regression model with CFS 1-4 as a reference.

Results

During the study period, 1,093 patients were triaged to our emergency department as needing tertiary care. After exclusion, 407 patients (median age = 80 years (interquartile range = 73-87)), were included in the study. Of these, 58 (14.2%) patients withdrew from intensive care within 24 hours. The adjusted odds ratios were 1.99 for CFS 5 (95% confidence interval (CI) = 0.74-5.32, p = 0.17), 2.48 for CFS 6 (95% CI = 0.93-6.57, p = 0.068), and 5.48 for CFS 7-9 (95% CI = 2.68-12.6, p < 0.01).

Conclusions

Higher CFS scores in critically ill older adults were associated with a higher likelihood of withdrawal within 24 hours. The goals-of-care conversation could be held proactively when CFS scores are high to reduce unwanted medical intervention.

## Introduction

Some older adults nearing the end of life would choose palliative care over intensive care if their functional capacity and quality of life (QOL) are severely compromised. Unfortunately, approximately 70% of patients in such situations are unconscious or unable to communicate their treatment preferences [1].

Advance care planning (ACP) is the process of preparing for future medical care in a way that respects a patient’s personal values, life goals, and wishes. ACP has demonstrated multiple benefits. It reduces hospitalizations, prevents unwanted intensive treatments at the end of life, and increases the likelihood that patients will pass away in their desired setting.

The completion rates for ACP are around 37% in the United States and 30% in Australia. In countries like the United Kingdom, Germany, and the Netherlands, the rates are roughly 8-10%. In Japan, while 70% of people believe it is important to express their end-of-life treatment preferences, only 2.6% use written advance directives [2,3]. This significant gap highlights the critical need for effective crisis communication in emergency departments to ensure patients receive the best possible care.

Typically, ACP involves discussions between patients, families, and healthcare providers over weeks, months, or even years. This process allows for trust and understanding to develop, resulting in treatment plans that honor the patient’s priorities. However, in the emergency department, such prolonged discussions are rarely possible. The sudden and urgent nature of emergencies leaves families with little time to make well-informed decisions consistent with the patient’s preferences. If we could identify in advance which patients are more likely to transition to palliative care, healthcare providers could dedicate more time to exploring their values and determining if their goals are achievable through medical intervention.

The Clinical Frailty Scale (CFS) is a scoring system initially developed by the Canadian Study of Health and Aging to assess the overall fitness or frailty of older adults [4]. The CFS has evolved into a widely used clinical tool for screening frailty and categorizing degrees of fitness. It correlates strongly (r = 0.80) with the Frailty Index and shows that each one-category increase significantly raises the medium-term risks of death and institutionalization. Although prior studies have examined the relationship between CFS and mortality, none have focused on withdrawal of care as a primary endpoint. The simplicity of the CFS makes it suitable for pre-hospital assessments, even by those with limited medical training, enabling families and emergency medical personnel to estimate a patient’s frailty score effectively.

This study aimed to explore whether it is possible to predict, before hospital transfer, which patients are more likely to transition to withdrawal of care and comfort-focused measures instead of invasive procedures. The CFS score was investigated as a potential tool for identifying these patients.

This article was previously presented as a meeting abstract at the American Geriatrics Society Annual Scientific Meeting on May 13, 2022.

## Materials and methods

Setting and design

This study was a retrospective, observational, single-center cohort study conducted between February 1, 2021, and January 31, 2022.

Patient population

Patients aged over 65 who were triaged to our tertiary care emergency department as needing tertiary-level care and subsequently admitted for further treatment were included. Triage was conducted by the Kobe emergency medical services. Although a triage protocol exists as a benchmark, it is primarily based on vital signs; therefore, emergency medical services often incorporate additional factors, such as patient history and physical examination, when triaging.

Out-of-hospital cardiac arrests (OHCAs) and “hot-line” cases (patients with suspected acute stroke or acute coronary syndrome who are automatically triaged as needing tertiary care) were excluded. Patients without reachable surrogates or those deemed to lack appropriate decision-making capacity were also excluded. Decision-making capacity was assessed based on the patient’s ability to understand, express a choice, appreciate, and reason [5].

Data collection

Multiple factors were assessed to evaluate whether the CFS could serve as a surrogate to identify patients more likely to transition to comfort measures. Factors considered included age, sex, CFS score, National Early Warning Score 2 (NEWS2), Charlson Comorbidity Index (CCI), and vital signs (consciousness, blood pressure, heart rate, respiratory rate, oxygen saturation, and temperature). Vital signs were recorded upon arrival, and NEWS2 and CCI were extracted and calculated via chart review.

CFS was determined by the attending emergency physician on arrival after consulting with the patient’s family to understand the patient’s usual lifestyle. The CFS scores range from 1 to 9: 1 (very fit), 2 (fit), 3 (managing well), 4 (living with very mild frailty), 5 (living with mild frailty), 6 (living with moderate frailty), 7 (living with severe frailty), 8 (living with very severe frailty), and 9 (terminally ill). Patients were categorized into the following three groups based on their CFS score: 1-4, 5-6, and 7-9.

Outcome measures

The primary outcome was discontinuation of treatment within 24 hours. This included cases where families chose to withdraw or withhold treatment within 24 hours after discussions with the emergency physician or the primary physician following admission. Withdrawal of treatment was defined as the cessation of life-sustaining therapy deemed ineffective or no longer appropriate. Withholding of treatment was defined as the decision not to initiate further therapeutic interventions.

Statistical measures

Descriptive statistics were reported as medians with interquartile ranges (IQRs) or means with standard deviations for continuous variables, and counts with proportions for categorical variables.

The primary analysis was conducted using the complete dataset, comparing outcomes across the three CFS groups, namely, 1-4 (fit to vulnerable), 5-6 (mildly to moderately frail), and 7-9 (severely frail to terminally ill). Adjusted risk differences and risk ratios were calculated using a logistic regression model, controlling for age (five-year increments), sex, NEWS2, and CCI. The association between CFS and discontinuation of treatment was examined, with CFS 1-4 used as the reference group.

Statistical analyses were performed using STATA, version 17.0 (StataCorp, College Station, TX, USA). All p-values were two-tailed, with a significance level of p < 0.05.

## Results

Study population

**Figure 1:**
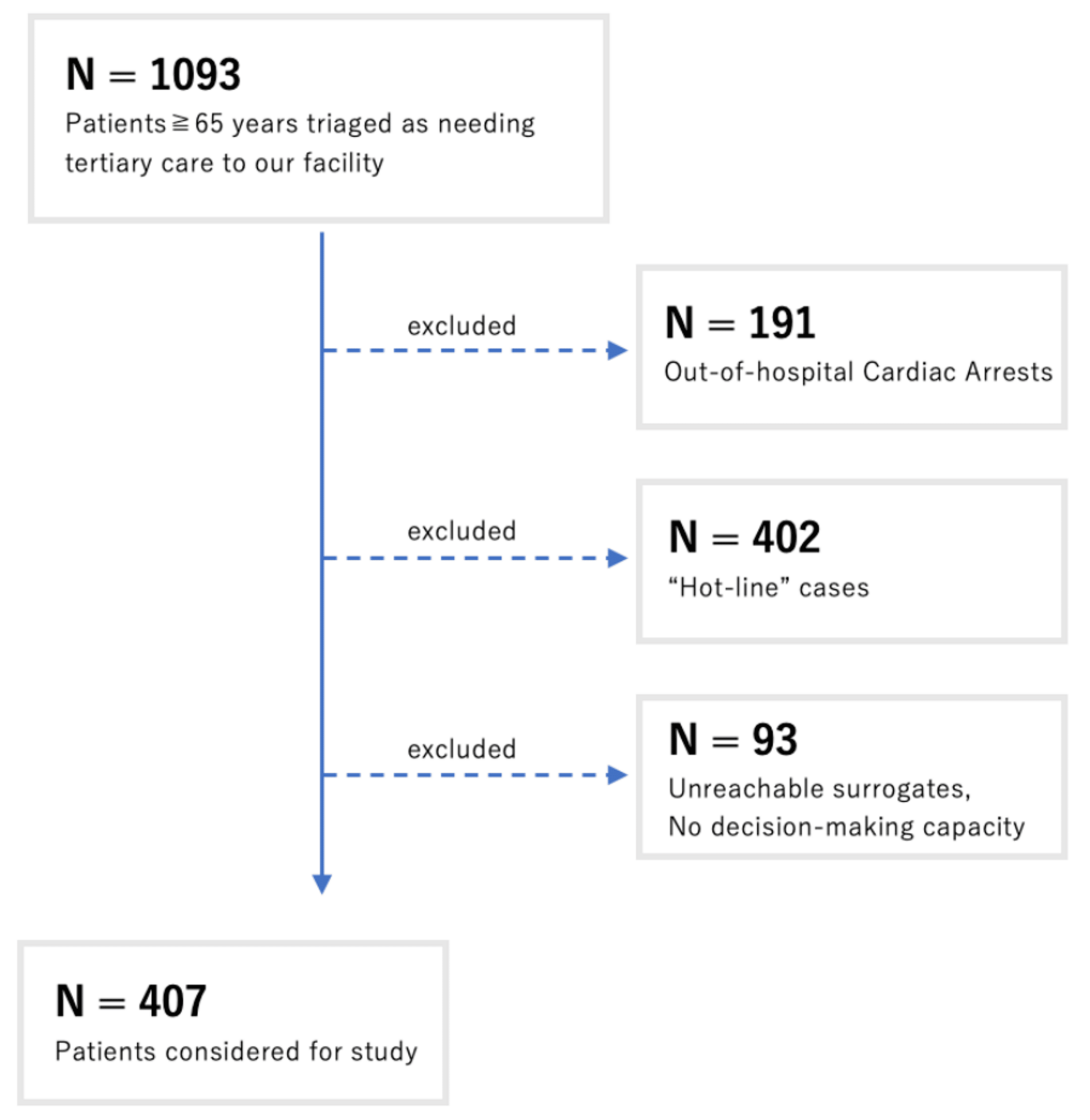
Patient flow.

Of the approximately 9,000 patients transported to our hospital, 1,093 patients aged over 65 were triaged as needing tertiary-level care. Of these, 191 were OHCAs, 402 were “hot-line” cases, and 93 patients either had no reachable surrogates or were deemed to lack appropriate decision-making capacity. After these exclusions, a total of 407 patients were included in the study (Figure 1).

Patient characteristics

The overall median age was 80 years (IQR = 73-87), with older patients more frequently categorized into higher (more severe) CFS groups. A higher percentage of females (43.5%) were observed in the less advanced CFS categories. Similarly, patients in lower CFS categories tended to present with more stable vital signs. The median vital signs were blood pressure of 128 mmHg (IQR = 94-160)/78 mmHg (IQR = 57-93), heart rate of 100 beats/minute (IQR = 80-120), respiratory rate of 33 breaths/minute (IQR = 24-37), and body temperature of 36.6℃ (IQR = 36.0-37.5). The overall median CCI score was 2.5 (IQR = 0-3.0), and the median NEWS2 was 9 (IQR = 7-11). Higher scores were observed in patients in more advanced CFS categories.

Main findings

Of the population identified, 58 (14.2%) patients discontinued treatment within 24 hours (Table 1).

**Table 1:** Baseline characteristics. CFS: Clinical Frailty Scale; IQR: interquartile range; SBP: systolic blood pressure; DBP: diastolic blood pressure; HR: heart rate; RR: respiratory rate; BT: body temperature; CCI: Charlson Comorbidity Index; NEWS2: National Early Warning Score 2

Characteristics	CFS 1-4	CFS 5	CFS 6	CFS 7-9	Total
N	181	71	67	88	407
Age, median (IQR)	76 (71-83)	83 (77-88)	82 (75-88)	84 (78-91)	80 (73-87)
Sex No. (%)					
Female	69 (38.1)	35 (49.3)	32 (47.8)	41 (46.6)	177 (43.5)
Vital signs, median (IQR)					
SBP (mmHg)	130 (100-165)	131(91-159)	121 (90-164)	118 (82-150)	128 (94-160)
DBP (mmHg)	80 (60-93)	74 (55-92)	80 (55-92)	72 (50-88)	78 (57-93)
HR (beats/minute)	95 (77.5-120)	100 (80-113)	106 (82-117)	105 (82-127)	100 (80-120)
RR (breaths/minute)	31 (24-36)	35 (25-37)	35 (24-36)	33 (28-37)	33 (24-37)
BT (℃)	36.5 (35.9-37.1)	36.5 (35.8-37.0)	36.8 (36.2-37.7)	36.9 (36.6-38.1)	36.6 (36-37.5)
CCI	1 (0-2)	2 (1-4)	2 (1-3)	2 (0-3)	2.5 (0-3)
NEWS2	8 (6-10)	9 (7-11)	9 (7-11)	10 (8-11)	9 (7-11)

When stratified to their respective CFS scores, unadjusted odds ratios were 2.24 for CFS 5 (95% confidence interval (CI) = 0.88-5.67, p = 0.09), 3.09 for CFS 6 (95% CI = 1.27-7.52, p = 0.013), and 6.95 for CFS 7-9 (95% CI = 3.25-14.88, p < 0.01) (Table 2).

**Table 2:** Primary outcomes. *: p-values <0.05 are statistically significant. CFS: Clinical Frailty Scale; CI: confidence interval

Characteristics	Odds ratio (95% CI)	P-value
CFS 1-4	Reference	-
CFS 5	2.24 (0.88-5.67)	0.09
CFS 6	3.09 (1.27-7.52)	0.013*
CFS 7-9	6.95 (3.25-14.88)	<0.01*

The adjusted odds ratios were 1.99 for CFS 5 (95% CI = 0.74-5.32, p = 0.17), 2.48 for CFS 6 (95% CI = 0.93-6.57, p = 0.068), and 5.48 for CFS 7-9 (95% CI = 2.68-12.6, p < 0.01) (Table 3).

**Table 3:** Multivariate analysis of primary outcomes. *: p-values <0.05 are statistically significant. CFS: Clinical Frailty Scale; CI: confidence interval; CCI: Charlson Comorbidity Index; NEWS2: National Early Warning Score 2

Characteristics	Odds ratio (95% CI)	P-value
CFS 1-4	Reference	-
CFS 5	1.99 (0.74-5.33)	0.17
CFS 6	2.48 (0.93-6.59)	0.068
CFS 7-9	5.48 (2.38-12.6)	<0.001*
Age (5-year increments)	1.34 (1.09-1.63)	0.005*
Female	1.25 (0.68-2.32)	0.47
CCI	0.59 (0.41-0.86)	0.005*
NEWS2	1.04 (0.57-1.90)	0.12

Although for CFS 5 and 6 the odds ratio was not statistically significant, for those stratified into the CFS 7-9 group, there was an increased likelihood of withholding or withdrawing treatment within 24 hours.

## Discussion

In this study, we explored the relationship between treatment discontinuation and the CFS. Our findings demonstrated that patients with higher CFS scores were significantly more likely to have treatment withdrawn or withheld within 24 hours. While limited research exists on ACP, goals-of-care discussions, and withdrawal rates in the emergency department, previous studies have investigated CFS as a predictor of long-term mortality in surgical wards, intensive care units (ICUs), and emergency settings. Wozniak et al. demonstrated that ICU patients with higher CFS scores were associated with increased mortality and poorer physical health-related QOL, findings that align with our study [6]. Similarly, Inaba et al. reported comparable results. However, these findings were largely confined to the ICU, and the role of CFS as a surrogate marker for initiating palliative care in the emergency department remains underexplored [7].

A scoping review of CFS concluded that it predicts outcomes in 74% of cases, with mortality being the most common outcome, where it was predictive in 87% of cases [8]. Our findings align with this, suggesting that frail patients often result in treatment withdrawal or withholding.

Despite the established value of frailty as a prognostic tool, the concept of integrating goals-of-care discussions into emergency departments remains underdeveloped. Japan’s ACP rates lag significantly behind Western nations, with only 20% of physicians trained in end-of-life discussions that respect patient autonomy [9]. In contrast, this figure is 70% in the United States [10]. Furthermore, Japan’s training predominantly targets general hospitalists rather than emergency physicians, indicating an even lower preparedness among emergency department staff. This disparity is not solely due to cultural differences but also systemic issues, such as the lack of incentives; for instance, U.S. Medicare began reimbursing ACP discussions in 2016. Yet, even in the United States, the emergency department remains underutilized for these critical conversations [11].

The issue is not a lack of interest or recognition of ACP’s value. A cross-sectional survey revealed that while 86.9% of palliative care physicians considered ACP an effective means for patients to express their medical preferences, only 30.3% practiced it regularly [12]. This highlights a significant gap between awareness and implementation, driven largely by a lack of training and confidence. In the emergency department, the challenge is compounded by time constraints and the difficulty of building rapport with patients and families during crises.

Incorporating the CFS system could help physicians identify patients at risk of receiving unwanted invasive treatments and prioritize discussions that better reflect patient values. A systematic review [13] suggested that ACP could reduce emergency department visits. Moreover, initiating goals-of-care conversations in the emergency department, even for patients who are ultimately discharged, might enable them to spend their remaining time at home or with loved ones.

Study limitations

This study has several limitations. First, we could not assess how the CFS influenced physicians’ decision-making during goals-of-care discussions. Higher CFS scores may inherently bias physicians toward withdrawal, potentially affecting the objectivity of our findings. Second, the COVID-19 pandemic likely influenced the accuracy of CFS scoring, as families often avoided close contact with patients to reduce infection risk, potentially leading to under-triaging. Finally, as a single-center retrospective study, our findings may not be generalizable. While factors such as severity and pre-existing conditions predictably contribute to outcomes, we accounted for confounding variables by incorporating NEWS and CCI scores to minimize their impact.

## Conclusions

Patients with higher CFS scores were significantly more likely to transition to comfort-focused measures, emphasizing the scale’s potential to guide early and meaningful goals-of-care discussions in the emergency department. However, challenges such as limited training in ACP, time constraints in emergency settings, and systemic barriers, particularly in Japan, hinder the integration of such discussions. By leveraging the simplicity of the CFS to identify frail patients at risk of receiving unwanted invasive treatments, healthcare providers can prioritize crisis communication and align care with patient values, ultimately enhancing the quality of end-of-life care.
